# Concentric ballooned catheterization to the fractional non-newtonian hybrid nano blood flow through a stenosed aneurysmal artery with heat transfer

**DOI:** 10.1038/s41598-021-99499-z

**Published:** 2021-10-14

**Authors:** Obaid Ullah Mehmood, Sehrish Bibi, Dzuliana F. Jamil, Salah Uddin, Rozaini Roslan, Mohd Kamalrulzaman Md Akhir

**Affiliations:** 1 Department of Mathematics, COMSATS University Islamabad, Wah Campus, Wah Cantt., 47040 Pakistan; 2 Faculty of Applied Sciences and Technology, Universiti Tun Hussein Onn Malaysia, Pagoh Campus, 84600 Muar, Johor Malaysia; 3 Department of Physical and Numerical Sciences, Qurtuba University of Science and Information Technology, Peshawar, 25000 Pakistan; 4 Institute of Engineering Mathematics, Faculty of Applied and Human Sciences, Universiti Malaysia Perlis, Pauh Putra Campus, 02600 Arau, Perlis Malaysia; 5 ANNA Systems LLC, Moscow Region, Dubna, 9 Maya Street, Building 7B, Building 2 Office 10.141707, Moscow, Dolgoprudnenskoe Highway, 3, Fiztekhpark, Moscow, 141980 Russia

**Keywords:** Computational biology and bioinformatics, Diseases, Mathematics and computing

## Abstract

The current work analyzes the effects of concentric ballooned catheterization and heat transfer on the hybrid nano blood flow through diseased arterial segment having both stenosis and aneurysm along its boundary. A fractional second-grade fluid model is considered which describes the non-Newtonian characteristics of the blood. Governing equations are linearized under mild stenosis and mild aneurysm assumptions. Precise articulations for various important flow characteristics such as heat transfer, hemodynamic velocity, wall shear stress, and resistance impedance are attained. Graphical portrayals for the impact of the significant parameters on the flow attributes have been devised. The streamlines of blood flow have been examined as well. The present finding is useful for drug conveyance system and biomedicines.

## Introduction

Studies related to arterial stenosis and arterial aneurysm have gained significant attention due to their recurrent occurrence in both young grown-ups and pediatric patients. The excess of certain nutrients such as cholesterol and fat can result in blockage in blood arteries. The constriction of an artery or heart valve disturbing the normal bloodstream is known as stenosis and the associated disease is known as arteriosclerosis. Arteriosclerosis occurs as the arteries turn out to be thick and more rigid, thus causing coronary artery infections, myocardial infarction, strokes, angina, and cardiac arrests. On the other hand, aneurysm is the expansion of an artery brought about by frailty in the arterial wall. Blood flow through veins turns out to be more confounded as aneurysm develops. Catheterization is now the most standard medical method for diagnosing and treating arterial diseases. A catheter is a dainty, empty cylinder that is injected into the vein. Catheters have been widely used in the medication of heart disease. During catheterization, little cylinders (catheters) are embedded into the circulatory framework under the X-ray direction to attain bloodstream data and pressing factors inside the heart, and to decide whether there are impediments inside the veins taking care of the heart muscle. Also, a catheter is utilized for the estimation of different physiological stream attributes, for example, pressure gradient and flow speed/stream rate. Moreover, catheters can be utilized for intermittent blood vessel and blood gas examinations in patients with respiratory disappointment, or extreme acid/base aggravation. At the point when a patient has a serious lung issue where regular checking of oxygen or carbon dioxide levels in the blood stream is required, catheter can be used without jabbing a patient on a consistent basis. When a catheter is embedded into a vein, blood clusters are formed at the tips of catheter thus obstructing the bloodstream. Besides, draining can happen as catheter is embedded. In any case, catheter infusion changes the hemodynamic conditions in the corridor. It tends to be utilized to maximize the supply of blood to indispensable organs. When a blood vessel is narrowed or blocked, a catheter with a balloon can be used to expand the vein so that the blood flow rate is increased. The study of blood circulation via catheterized stenotic aneurysmal arteries is gaining prominence, owing to the ever-increasing requirements of science and medicine.

The mathematical modeling and numerical simulations of an unsteady axisymmetric blood flow through a porous diseased arterial section were presented by Mehmood et al.^1^. Nadeem and Ijaz^2^ investigated the non-isothermal, viscous blood flow through inclined arteries having both stenosis and dilatation. The blood flow through a stenosed channel was studied by Akbar and Nadeem^3^. Mild stenosis on blood flow was typically regarded as pivotally non-symmetric but radially symmetric. Blood was considered as Williamson liquid and the flow was treated as steady. Shit and Majee^4^ investigated numerically, the flow pattern in a diseased artery segment with abdominal aortic aneurysm by considering both unsteady magneto-hydrodynamic (MHD) bloodstream and heat transfer models. The thermal energy condition was examined by taking into account the energy dissipation owing to the applied magnetic field and blood viscosity. Abdelsalama et al.^5^ investigated the physical features of electro-magneto-hydrodynamics (EMHD) in the context of electroosmotic forces on a diseased artery section with both stenosis and aneurysm. Numerous experts have studied the blood flow streams in regular, aneurysmal, and stenotic arteries^6–9^. Mekheimer and El Kot^10^ modeled a surgical procedure where catheters were inserted into stenotic arteries. The physiological Newtonian fluid moved between two eccentric cylinders. The numerical study of Jeffrey liquid with nanoparticles flowing within the diseased atherosclerotic segment was undertaken by Ellahi et al.^11^. In addition, the convective heat exchange with a catheter was taken into account. Reddy et al.^12^ investigated the incompressible and homogenous coupled-stress blood flow via a catheterized diseased stenotic tapered artery. Elnaqeeb et al.^13^ examined the bloodstream with copper nanoparticles in a catheterized stenosed artery with thrombosis. Under mild stenosis condition, the coupled governing conditions were characterized and rearranged. Misra et al.^14^ investigated the flow pattern of non-Newtonian blood in a stenosed artery when a catheter was inserted. The Herschel-Bulkley fluid model was used to describe the rheology of blood. Various assessments on the effect of expansion of catheters in the presence of stenosis and aneurysm on the bloodstream have been performed^15–17^.

Nanotechnology centers around miniature items and the creation of the issue. The size of nanoparticle is ~ 100 nm. The innate capacity of nanotechnology permits the transport of prescriptions to various segments of the human body empowering a proficient conveyance of cargo inside tissues and cells. Nanotechnology is acclaimed as having the capacity to build proficiency in energy utilization and tackle significant medical conditions. The result of nanotechnology is more modest, more practical, and entails less energy. Nanotechnology has opened a scholarly field of science alongside its applications. New attributes may likewise be found in nanoparticles, for example, gold and copper nanoparticles. As an outcome, these most recent advances have critical clinical notoriety in medication. Another type of nanofluids is called as hybrid nanofluid, which is made of two or more nanomaterials. Adjoining nanoparticles to base liquid could enhance the heat transmission quality of base liquid. A hybrid nanoparticle is an extraordinary compound and it has been endlessly utilized in the production of anti-cancer medications. Maskeen et al.^18^ investigated the heat transfer and stream characteristics of alumina–copper/water hybrid nanofluid over an expanding tube subjected to Lorentz's forces and warm radiation. Mekheimer et al.^19^ studied the non-isothermal, non-newtonian blood flow pattern between two coaxial tubes containing gold nanoparticles. In order to find an empirical solution of the velocity profile, the normal perturbation approach was applied. An investigation of bio-nanofluid with copper in adaptable walls was performed by Shahzadi et al.^20^. Hypothetical examinations on single nanofluid and hybrid nanofluid can be found in^21–30^.

For blood vessel of width less than 0.5 mm, blood possesses limited yield tension and shear dependent viscosity. Further, the blood stream is partitioned into two stages of a cell-rich focus zone and a fringe plasma zone. These features separate speculations that are significant for the derivation of Newtonian fluid governing equations. The treatment of blood as Newtonian fluid may not be valid as coronary courses have a distance across of less than 0.5 mm. Accordingly, the viscoelastic partial model is selected to depict the blood development through the coronary veins. The final mathematical model is obtained by modifying the ordinary time-derivative differential conditions into the fractional time-derivative ones. Fractional math has been used to manage distinctive rheological problems. Of a couple of models proposed for physiological liquids, the fractional second-grade liquid model is popular although this model limits the fractional time-inferred boundary to a second-grade work ($\alpha$ = 0). Furthermore, the Navier–Stokes model can be formulated by putting the second-grade material constant $\lambda_{1} = 0$.

Maiti et al.^31^ investigated the capture productivity of magnetic nanoparticles combined with therapeutic compounds injected into the bloodstream which were monitored using an outer magnetic field. With a non-Newtonian bi-viscosity fluid, the thermo-solutal transfer with Caputo-Fabrizio fractional-order derivative is demonstrated. Uddin et al.^32^ examined the heat transfer phenomena and the influence of a magnetic field on the second-grade fluid in an upward oscillating cylinder. The fluid is magnetized by adding a perpendicular magnetic field. The Caputo-Fabrizio non-integer derivative approach was utilized to simulate fractional MHD flow. Maiti et al.^33^ proposed a fractional-order time derivative model for simulating blood flow, mass and heat transfers along a blood vessel subjected to magnetic field. They used the non-Newtonian Casson liquid model to investigate the unidirectional bloodstream in permeable medium vessels. The slip impact on the peristaltic flow of a fractional second-grade fluid through a round and hollow cylinder was studied by Rathod and Tuljappa^34^. Also, they have adopted the Caputo-Fabrizio fractional derivative. More details on the fractional fluid model can be found in^35–38^.

Taking into account the previously mentioned research articles, the main aim of this research is to investigate the combined effects of hybrid fractional second-grade fluid model and concentric catheterization in a diseased artery having stenosis and aneurysm with heat transfer. To the best of our knowledge, a study of this kind has not been performed before. For mild stenosis and mild aneurysm situations, the problem has been simplified and the exact solutions of temperature, hemodynamic velocity, wall shear stress, and resistance impedance for the flow can have been obtained. Various important flow phenomena are unearthed upon examining the plotted graphs and streamlines. Finally, the main findings of the outcomes are summarized.

## Problem formulation

We consider the fractional second-grade, non-isothermal, hybrid nano-blood flow in an annular region bounded by two coaxial tubes. The outer tube contains an axially symmetric mild stenosis and aneurysm while the inner tube represents the catheter. Balloon catheterization is accomplished in the stenotic segment. We deal with the cylindrical coordinate system $(r,\theta ,z)$ where the *z*-axis is taken as the axis of the artery. The geometries of the outer and the inner walls are defined by^5,13^:

1 $$ R(\overline{z}) = \left\{ {\begin{array}{*{20}c} {1 - \frac{\delta }{2b}\left( {1 + \cos \frac{2\pi }{{L_{j} }}\left( {\overline{z} - \beta_{j} - \frac{{L_{j} }}{2}} \right)} \right),} & {\beta_{j} \le \overline{z} \le \beta_{j} + L_{j} ,\;\;\; \, j = 1,2} \\ { \, 1} & {(otherwise),} \\ \end{array} } \right. $$

2 $$ \chi (\overline{z}) = \left\{ {\begin{array}{*{20}c} {b\left( {e + \sigma Exp( - \pi^{2} (\overline{z} - z_{d} - 0.5)^{2} )} \right),} & {\beta_{1} \le \overline{z} \le \beta_{1} + L_{1} ,} \\ {e \, b} & {(otherwise),} \\ \end{array} } \right. $$

where the length of the *j*th irregular stenosed section emanating from the origin is $\beta_{j}$, the normal arterial radius is denoted by $b$, the length of the *j*th irregular stenosed section is represented by $L_{j}$, and the critical height of the *j*th irregular stenosed section is denoted by $\delta$. It is important to mention here that $\delta$ is positive for stenosis and negative for an aneurysm. Two specific positions of the critical heights of $\delta$ are

3 $$ \overline{z}_{1} = \beta_{1} + \frac{{L_{1} }}{2}{,}\;\;\;{\text{and}}\;\;\;\overline{z}_{2} = \beta_{2} + \frac{{L_{2} }}{2}, $$

For the sake of simplicity, we have assumed $L_{1} = L_{2} = L_{0}$. $\sigma$ is the catheter’s maximum height at $\overline{z}$ = $z_{d}$ + 0.5 and $e \, b$ is the inner radius of the catheter. Here, $e$ is very small and $z_{d}$ is the axial displacement of the balloon during catheterization.

For fractional derivate, we use the Caputo’s definition defined as^5,34^:

4 $$ D^{\alpha } f(t) = \frac{1}{\Gamma (1 - \alpha )} \, \int_{h}^{t} { \, \frac{{f^{m} (\tau )}}{{(t - \tau )^{\alpha + 1 - m} }}} \, d\tau ,\;\;\;(m - 1 < {\text{Re}} \, (\alpha ) \le m, \, m \in N), $$

where $\alpha$ is the order of the derivative and *h* is the initial guess of $f$. For the derivatives, we will use Caputo’s derivative condition defined as^5,34^:

5 $$ D^{\alpha } t^{\beta } = \begin{array}{*{20}c} 0 & {for \, (\beta \le \alpha - 1)} \\ {\frac{\Gamma (\beta + 1)}{{\Gamma (\beta + 1 - \alpha )}}t^{\beta - \alpha } } & {for \, (\beta > \alpha - 1).} \\ \end{array} $$

**Figure 1:**
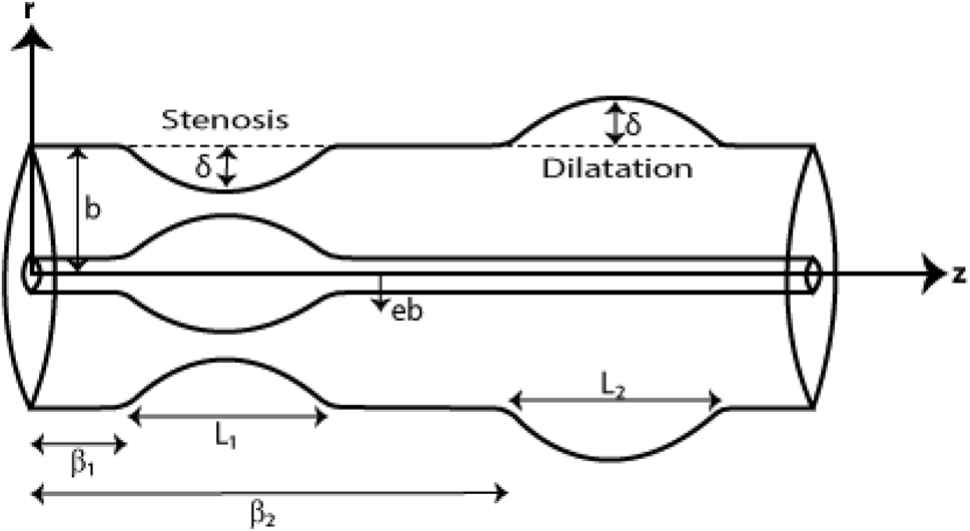
Configuration of the physical model.

The physical configuration of the blood flow under consideration is given in Fig. 1.

The relationship between fractional second-grade and viscoelastic fluids can be found in^5^:

6 $$ \begin{gathered} \overline{S} = \mu \left( {1 + \overline{\lambda }_{1} \left( {\frac{\partial }{{\partial \overline{t}}}} \right)^{\alpha } } \right)\dot{\gamma }, \hfill \\ {\text{where}}\,\alpha = \left\{ {\begin{array}{*{20}c} 0 & {{\text{Ordinary}}\;{\text{second - grade}}\;{\text{fluid}}} \\ { < 1} & {{\text{Fractional}}\;{\text{second - grade}}\;{\text{fluid}}} \\ \end{array} } \right. \hfill \\ \end{gathered} $$

In the above expression, $\mu$ is viscosity, $\overline{t}$ is time, $\overline{S}$ is shear stress, $\overline{\lambda }_{ \, 1}$ is material constant, $\dot{\gamma }$ is shear strain rate and $\alpha \, (0 \le \alpha \le 1)$ is the time-derivative fractional parameter. The fractional second-grade model coincides with the ordinary second-grade model if $\alpha$ = 1 and it reduces to a Navier–Stokes model if $\overline{\lambda }_{1} = 0$. Along with gravity (body force), the scalar forms of the governing equations for axisymmetric flow with the fractional second-grade model for viscoelastic fluid are as follows:

7 $$ \frac{{\partial \overline{u}}}{{\partial \overline{r}}} + \frac{{\overline{u}}}{{\overline{r}}} + \frac{{\partial \overline{w}}}{{\partial \overline{z}}} = 0, $$

8 $$ \rho_{hnf} \left( {\frac{{\partial \overline{u}}}{{\partial \overline{t}}} + \overline{u}\frac{{\partial \overline{u}}}{{\partial \overline{r}}} + \overline{w}\frac{{\partial \overline{u}}}{{\partial \overline{z}}}} \right) = - \frac{{\partial \overline{p}}}{{\partial \overline{r}}} + \mu_{hnf} \left( {1 + \overline{\lambda }_{1}^{\alpha } \left( {\frac{\partial }{{\partial \overline{t}}}} \right)^{\alpha } } \right)\left[ {\frac{{\partial^{2} \overline{u}}}{{\partial \overline{r}^{2} }} + \frac{1}{{\overline{r}}}\frac{{\partial \overline{u}}}{{\partial \overline{r}}} + \frac{{\partial^{2} \overline{u}}}{{\partial \overline{z}^{2} }} - \frac{{\overline{u}}}{{\overline{r}^{2} }}} \right], $$

9 $$ \rho_{hnf} \left( {\frac{{\partial \overline{w}}}{{\partial \overline{t}}} + \overline{u}\frac{{\partial \overline{w}}}{{\partial \overline{r}}} + \overline{w}\frac{{\partial \overline{w}}}{{\partial \overline{z}}}} \right) = - \frac{{\partial \overline{p}}}{{\partial \overline{z}}} + \mu_{hnf} \left( {1 + \overline{\lambda }_{1}^{\alpha } \left( {\frac{\partial }{{\partial \overline{t}}}} \right)^{\alpha } } \right)\left[ {\frac{{\partial^{2} \overline{w}}}{{\partial \overline{r}^{2} }} + \frac{1}{{\overline{r}}}\frac{{\partial \overline{w}}}{{\partial \overline{r}}} + \frac{{\partial^{2} \overline{w}}}{{\partial \overline{z}^{2} }}} \right] + (\rho \gamma )_{hnf} g(\overline{T} - \overline{T}_{0} ), $$

10 $$ (\rho c)_{hnf} \left( {\frac{{\partial \overline{T}}}{{\partial \overline{t}}} + \overline{u}\frac{{\partial \overline{T}}}{{\partial \overline{r}}} + \overline{w}\frac{{\partial \overline{T}}}{{\partial \overline{z}}}} \right) = k_{hnf} \left( {\frac{{\partial^{2} \overline{T}}}{{\partial \overline{r}^{2} }} + \frac{1}{{\overline{r}}}\frac{{\partial \overline{T}}}{{\partial \overline{r}}} + \frac{{\partial^{2} \overline{T}}}{{\partial \overline{z}^{2} }}} \right) + Q_{0} , $$

where $\overline{r}$ represents the radial direction and $\overline{z}$-axis is taken along the axis of the artery. $\overline{u}$ and $\overline{w}$ are radial and axial velocity components, $Q_{ \, 0}$ is constant of heat generation, $\mu_{hnf}$, $k_{hnf}$, $\rho_{hnf}$, $(\rho c)_{hnf}$ and $\gamma_{hnf}$ are viscosities, thermal conductivities, densities, heat capacitances, and thermal expansions of the hybrid nanoparticles. The electric conductivity of hybrid nanoparticle is denoted as $\sigma_{hnf}$. $\overline{p}$ is the fluid pressure and $\overline{T}$ is the fluid temperature.

The appropriate dimensional boundary conditions are^13^:

11 $$ \begin{gathered} \overline{w} = 0\;\;\;{\text{at}}\;\;\;r = \chi (\overline{z})\;\;\;{\text{and}}\;\;\;\overline{w} = 0\;\;\;{\text{at}}\;\;\;r = R(\overline{z}), \hfill \\ \overline{T} = \overline{T}_{1} \;\;\;{\text{at}}\;\;\;r = \chi (\overline{z})\;\;\;{\text{and}}\;\;\;\overline{T} = \overline{T}_{0} \;\;\;\;{\text{at}}\;\;\; \, r = R(\overline{z}). \hfill \\ \end{gathered} $$

where $T_{1}$ is the temperature of the catheter wall and $T_{0}$ is the temperature of the arterial wall. Thermophysical properties of nano blood flow are described as^5^

12 $$ \begin{aligned} \frac{{\mu_{nf} }}{{\mu_{f} }} & = \frac{1}{{\left( {1 - \phi } \right)^{2.5} }},\;\;\;\left( {\rho c} \right)_{nf} = (1 - \phi )\left( {\rho c} \right)_{f} + \, \phi \left( {\rho c} \right)_{s} , \, \\ \left( {\rho \gamma } \right)_{nf} & = (1 - \phi )\left( {\rho \gamma } \right)_{f} + \phi \left( {\rho \gamma } \right)_{s} , \, \rho_{nf} = (1 - \phi )\rho_{f} + \phi \rho_{s} , \, \\ \frac{{k_{nf} }}{{k_{f} }} & = \frac{{k_{s} + 2k_{f} - 2\phi \left( { - k_{f} + k_{s} } \right)}}{{k_{s} + 2k_{f} - \phi \left( { - k_{f} + k_{s} } \right)}}. \\ \end{aligned} $$

Thermophysical properties of hybrid nano blood flow are described as^5^

13 $$ \begin{aligned} \frac{{\mu_{hnf} }}{{\mu_{f} }} & = \frac{1}{{\left( {1 - \phi_{1} } \right)^{2.5} \left( {1 - \phi_{2} } \right)^{2.5} }}, \\ \frac{{\left( {\rho \gamma } \right)_{hnf} }}{{\left( {\rho \gamma } \right)_{f} }} & = \left( {\left( {1 - \phi_{2} } \right)\left( {\left( {1 - \phi_{1} } \right) + \phi_{1} \frac{{\left( {\rho \gamma } \right)_{s1} }}{{\left( {\rho \gamma } \right)_{f} }}} \right) + \phi_{2} \frac{{\left( {\rho \gamma } \right)_{s2} }}{{\left( {\rho \gamma } \right)_{f} }}} \right), \\ \frac{{k_{hnf} }}{{k_{bf} }} & = \frac{{k_{s2} + \left( {n - 1} \right)k_{bf} - \left( {n - 1} \right)\phi_{2} \left( {k_{bf} - k_{s2} } \right)}}{{k_{s2} + (n - 1)k_{bf} + \phi_{2} \left( {k_{bf} - k_{s2} } \right)}}, \\ \frac{{k_{bf} }}{{k_{f} }} & = \frac{{k_{s1} + \left( {n - 1} \right)k_{f} - \left( {n - 1} \right)\phi_{1} \left( {k_{f} - k_{s1} } \right)}}{{k_{s1} + (n - 1)k_{f} + \phi_{1} \left( {k_{f} - k_{s1} } \right)}}. \\ \end{aligned} $$

where $\mu_{f}$, $\rho_{f}$, $\left( {\rho c} \right)_{f}$, $k_{f}$ and $\gamma_{f}$ are viscosity, density, heat capacitance, thermal conductivity, and thermal expansion of the base fluid, respectively. Similarly, $\rho_{s1}$ and $\rho_{s2}$ , $\gamma_{s1}$ and $\gamma_{s2}$ , $k_{s1}$ and $k_{s1}$ are densities, thermal expansions, thermal conductivities of the hybrid nanoparticles respectively. $\phi_{1} {\text{ and }}\phi_{2}$ are the volume fractions of nanoparticles and $n$ is the shape factor of nanoparticles.

A distinction between hybrid nanofluid and nanofluid is made. Table 1 provides the thermophysical data for blood containing $Au$ and $Al_{2} O_{3}$ nanoparticles. Table 2 shows the shapes of nanoparticles and their respective shape factors.

**Table 1:** Thermophysical properties of hybrid nanoparticles^5^.

Physical properties	Fluid phase (*f*)	Nanoparticles phases ($s$)	
		Solid phase ($s_{1}$)	Solid phase ($s_{2}$)
	Blood	Au	${\text{Al}}_{{2}} {\text{O}}_{{3}}$
$\gamma$ (1/K)	0.18	1.42	8.9
$k$ (W/mk)	0.492	400	40
$\rho$ (kg/m^3^)	1063	19,300	3970

**Table 2:** Nanoparticles shapes with their shape factors^5^.

Shapes	Platelets	Cylinders	Bricks	Spheres
Shape factors	5.7	4.9	3.7	3

## Mild disease approximations

We define the following variables for the non-dimensionalization

14 $$ \begin{aligned} r & = \frac{{\overline{r}}}{b},\;\;\;w = \frac{{\overline{w}}}{U},\;\;\;z = \frac{{\overline{z}}}{{L_{j} }},\;\;\;u = \frac{{L_{j} \overline{u}}}{U\delta },\;\;\;p = \frac{{b^{2} }}{{UL_{j} \mu_{f} }}\overline{p},\;\;\;t = \frac{{U\overline{t}}}{{L_{j} }},\;\;\;\lambda_{1} = \frac{{U\overline{\lambda }_{1} }}{{L_{j} }}, \\ G_{r} & = \frac{{(\rho \gamma )_{f} gb^{2} (\overline{T}_{1} - \overline{T}_{0} )}}{{\mu_{f} U}},\;\;\;Q = \frac{{Q_{0} b^{2} }}{{\left( {\overline{T}_{1} - \overline{T}_{0} } \right)k_{f} }},\;\;\;\theta = \frac{{\overline{T} - \overline{T}_{0} }}{{\overline{T}_{1} - \overline{T}_{0} }}. \\ \end{aligned} $$

where $U$ is defined as the averaged velocity over the tube section of radius $b$. The temperature is expressed by $\theta$, heat source parameter is denoted by $Q$ and $G_{r}$ is the Grashof number. Under the assumptions of mild stenosis and mild aneurysm, we apply the following conditions^5^

15 $$ a = \frac{\delta }{b} < < 1, $$

16 $$ \Omega = \frac{b}{{L_{j} }} \approx 0(1). $$

The linearized Eqs. (7) to (10) take the following forms

17 $$ \frac{\partial p}{{\partial r}} = 0, $$

18 $$ \frac{\partial p}{{\partial z}} = \frac{{\mu_{hnf} }}{{\mu_{f} }}\left( {1 + \lambda_{1}^{\alpha } \left( {\frac{\partial }{\partial t}} \right)^{\alpha } } \right)\left[ {\frac{{\partial^{2} w}}{{\partial r^{2} }} + \frac{1}{r}\frac{\partial w}{{\partial r}}} \right] + \frac{{(\rho \gamma )_{hnf} }}{{(\rho \gamma )_{f} }}G_{r} \theta , $$

19 $$ \frac{{\partial^{2} \theta }}{{\partial r^{2} }} + \frac{1}{r}\frac{\partial \theta }{{\partial r}} + Q\frac{{k_{f} }}{{k_{hnf} }} = 0. $$

The dimensionless boundary conditions are

20 $$ \begin{gathered} w = 0\;\;\;{\text{at}}\;\;\;r = \chi (z)\;\;\;{\text{and}}\;\;\; \, w = 0\;\;\;{\text{at}}\;\;\;r = R(z), \hfill \\ \theta = 1\;\;\;{\text{at}}\;\;\;r = \chi (z)\;\;\;{\text{and}}\;\;\;\theta = 0\;\;\;{\text{at}}\;\;\;r = R(z). \hfill \\ \end{gathered} $$

The dimensionless forms of arterial and catheter walls $R(z)$ and $\chi (z)$ are

21 $$ R(z) = \left\{ {\begin{array}{*{20}c} {1 - \frac{a}{2}\left( {1 + \cos 2\pi \left( {z - \varepsilon_{j} - \frac{1}{2}} \right)} \right),} & {\varepsilon_{j} \le z \le \varepsilon_{j} + 1,} \\ 1 & {(otherwise),} \\ \end{array} } \right. $$

where $$\varepsilon_{j} = \frac{{\beta_{j} }}{{L_{j} }},\;and\;j = 1,2.$$

22 $$ \chi (z) = \left\{ {\begin{array}{*{20}c} {e + \sigma Exp\left( { - \pi^{2} L_{1}^{2} \left( {z - \frac{{z_{d} + 0.5}}{{L_{1}^{2} }}} \right)^{2} } \right),} & {\varepsilon_{1} \le z \le \varepsilon_{1} + 1,} \\ e & {(otherwise),} \\ \end{array} } \right. $$

## Solution of the problem

The general solution of temperature is determined from Eq. (19) and, by applying the corresponding temperature boundary conditions on arterial and catheter walls given in Eq. (20), the temperature is determined in the from.

23 $$ \theta (r) = - \frac{Q}{4}\frac{{k_{f} }}{{k_{hnf} }}\left( {r^{2} - R^{2} } \right) + \frac{{\ln \left( \frac{r}{R} \right)}}{{\ln \left( {\frac{\chi }{R}} \right)}}\left( {1 - \frac{Q}{4}\frac{{k_{f} }}{{k_{hnf} }}\left( {R^{2} - \chi^{2} } \right)} \right), $$

By utilizing the above calculated temperature and Eq. (13), the general solution of hemodynamic velocity has been obtained. Upon applying the corresponding conditions of hemodynamic velocity on arterial and catheter walls in Eq. (20), the analytical solution of the hemodynamic velocity has been obtained given by

24 $$ w(r) = \frac{1}{4c}\frac{dp}{{dz}}\frac{{\mu_{f} }}{{\mu_{hnf} }}\left( {r^{2} - R^{2} } \right) - \frac{1}{c}\frac{{\mu_{f} }}{{\mu_{hnf} }}\frac{{\left( {\rho \gamma } \right)_{hnf} }}{{\left( {\rho \gamma } \right)_{f} }}G_{r} A(r) + B\ln \left( \frac{r}{R} \right), $$

where $A(r)$ and $B$ are defined as

$$ A(r) = - \frac{Q}{4}\frac{{k_{f} }}{{k_{hnf} }}\left( {\frac{1}{16}r^{4} - \frac{1}{4}r^{2} R^{2} + \frac{3}{16}R^{4} } \right) + \frac{{\left( {1 - \frac{Q}{4}\frac{{k_{f} }}{{k_{hnf} }}\left( {R^{2} - \chi^{2} } \right)\left( {R^{2} - r^{2} + r^{2} \ln \left( \frac{r}{R} \right)} \right)} \right)}}{{4\ln \left( {\frac{\chi }{R}} \right)}}, $$

$$ B = \frac{{\frac{1}{4c}\frac{dp}{{dz}}\frac{{\mu_{f} }}{{\mu_{hnf} }}\left( {R^{2} - \chi^{2} } \right) + \frac{1}{c}\frac{{\mu_{f} }}{{\mu_{hnf} }}\frac{{\left( {\rho \gamma } \right)_{hnf} }}{{\left( {\rho \gamma } \right)_{f} }}G_{r} A(\chi )}}{{\ln \left( {\frac{\chi }{R}} \right)}},\;{\text{and}}\;c = 1 + \lambda_{1}^{\alpha } \left( {\frac{\partial }{\partial t}} \right)^{\alpha } . $$

## Validation

By considering nanofluid instead of hybrid nanofluid (i.e. $\phi_{2} = 0$ and $n = 3$), and by assuming the relaxation time parameter $\lambda_{ \, 1} = 0$, our results are in acceptable agreement with those reported by Elnaqeeb et al.^13^ (Eq. (19) and Eq. (20)) in the forms

25 $$ \theta (r) = - \frac{Q}{4}\frac{{k_{f} }}{{k_{nf} }}\left( {r^{2} - R^{2} } \right) + \frac{{\ln \left( \frac{r}{R} \right)}}{{\ln \left( {\frac{\chi }{R}} \right)}}\left( {1 - \frac{Q}{4}\frac{{k_{f} }}{{k_{nf} }}\left( {R^{2} - \chi^{2} } \right)} \right), $$

26 $$ w(r) = \frac{1}{4}\frac{dp}{{dz}}\frac{{\mu_{f} }}{{\mu_{nf} }}\left( {r^{2} - R^{2} } \right) - \frac{{\mu_{f} }}{{\mu_{nf} }}\frac{{\left( {\rho \gamma } \right)_{nf} }}{{\left( {\rho \gamma } \right)_{f} }}G_{r} A(r) + B\ln \left( \frac{r}{R} \right), $$

where $A(r)$ and $B$ are defined as

$$ A(r) = - \frac{Q}{4}\frac{{k_{f} }}{{k_{nf} }}\left( {\frac{1}{16}r^{4} - \frac{1}{4}r^{2} R^{2} + \frac{3}{16}R^{4} } \right) + \frac{{\left( {1 - \frac{Q}{4}\frac{{k_{f} }}{{k_{nf} }}\left( {R^{2} - \chi^{2} } \right)\left( {R^{2} - r^{2} + r^{2} \ln \left( \frac{r}{R} \right)} \right)} \right)}}{{4\ln \left( {\frac{\chi }{R}} \right)}}, $$

$$ B = \frac{{\frac{1}{4}\frac{dp}{{dz}}\frac{{\mu_{f} }}{{\mu_{nf} }}\left( {R^{2} - \chi^{2} } \right) + \frac{{\mu_{f} }}{{\mu_{nf} }}\frac{{\left( {\rho \gamma } \right)_{nf} }}{{\left( {\rho \gamma } \right)_{f} }}G_{r} A(\chi )}}{{\ln \left( {\frac{\chi }{R}} \right)}}. $$

## Significant flow characteristics

The dimensionless volume flow rate is expressed as

27 $$ F(z) = \int\limits_{\chi }^{R} {rw \, dr} . $$

The volume flow rate $F(z)$ can also be written in the form of linear combination of $S_{1}$ and $S_{2}$, where $S_{1}$ and $S_{2}$ are given below

28 $$ F(z) = S_{1} \frac{dp}{{dz}} + S_{2} , $$

29 $$ S_{1} = - \frac{1}{16c}\frac{{\mu_{f} }}{{\mu_{hnf} }}\frac{{\left( {\left( {R^{2} - \chi^{2} } \right)^{2} + \left( { - R^{4} + \chi^{4} } \right) \, \ln \left( R \right) + \left( {R^{4} - \chi^{4} } \right) \, \ln \left( \chi \right)} \right)}}{{\left( {\ln \left( {\frac{\chi }{R}} \right)} \right)}}, $$

30 $$ S_{2} = - \frac{1}{384c}\frac{{\mu_{f} }}{{\mu_{hnf} }}\frac{{\left( {\rho \gamma } \right)_{hnf} }}{{\left( {\rho \gamma } \right)_{f} }}\frac{{ \, G_{r} \left( \begin{gathered} - Q\left( {R^{2} - \chi^{2} } \right)\left( {6\left( {R^{2} - \chi^{2} } \right)^{2} - \left( \begin{gathered} - 9R^{4} + 9\chi^{4} - 4 \hfill \\ \left( {R^{4} + R^{2} \chi^{2} + \chi^{4} } \right)\left( {\ln \left( {\frac{\chi }{R}} \right)} \right) \hfill \\ \end{gathered} \right)\left( {\ln \left( {\frac{\chi }{R}} \right)} \right)} \right)k_{f} \hfill \\ + 6\left( {4\left( {R^{2} - \chi^{2} } \right)^{2} - \left( { - 3R^{4} - 4R^{2} \chi^{2} + 7\chi^{4} - 4\chi^{4} \left( {\ln \left( {\frac{\chi }{R}} \right)} \right)} \right)\left( {\ln \left( {\frac{\chi }{R}} \right)} \right)} \right)k_{hnf} \hfill \\ \end{gathered} \right)}}{{\left( {\ln \left( {\frac{\chi }{R}} \right)} \right)^{2} k_{hnf} \left( {\rho \gamma } \right)_{f} }}, $$

31 $$ \frac{dp}{{dz}} = \frac{{F - S_{2} }}{{S_{1} }}. $$

Since the stream rate $F$ is consistent in the annular region, the pressure rise and resistance impedance for non-dimensional arterial segment under consideration are given by

32 $$ \Delta p = \int\limits_{0}^{1} {\left( { - \frac{dp}{{dz}}} \right) \, } dz, $$

33 $$ \lambda = \frac{\Delta p}{F}. $$

Expressions for wall shear stress on arterial wall segment and catheter wall segment in dimensionless forms are given as

34 $$ \tau_{R} = \left. {\left( {\frac{\partial w}{{\partial r}}} \right)} \right|_{r = R(z)} , $$

35 $$ \tau_{\chi } = \left. {\left( {\frac{\partial w}{{\partial r}}} \right)} \right|_{r = \chi (z)} . $$

Stream function $\psi$ can be calculated by using $\frac{\partial \psi }{{\partial r}} = w * r$ and $\psi = 0$ at $r = \chi$. The stream function $\psi$ is calculated in the following manner

36 $$ \psi = \frac{1}{768c}\frac{{\mu_{f} }}{{\mu_{hnf} }}\frac{{\left( {G_{r} QD(r)k_{f} \left( {\rho \gamma } \right)_{hnf} + 12k_{hnf} \left( \begin{gathered} - 4\frac{dp}{{dz}}\left( {\ln \left( {\frac{\chi }{R}} \right)} \right)\left( \begin{gathered} 2r^{2} \left( { - R^{2} + \chi^{2} } \right)\ln \left( r \right) + \left( {r^{2} - \chi^{2} } \right) \hfill \\ \left( {R^{2} - \chi^{2} + \left( {r^{2} - \chi^{2} } \right)\ln \left( R \right)} \right) - \hfill \\ \left( {r^{4} - 2r^{2} R^{2} + \chi^{4} } \right)\ln \left( \chi \right) \hfill \\ \end{gathered} \right) \hfill \\ \left( {\rho \gamma } \right)_{f} + G_{r} E(r)\left( {\rho \gamma } \right)_{hnf} \hfill \\ \end{gathered} \right)} \right)}}{{\left( {\ln \left( {\frac{\chi }{R}} \right)} \right)^{2} k_{hnf} \left( {\rho \gamma } \right)_{f} }} $$

where $D(r)$ and $E(r)$ are defined as

$$ D(r) = \left( \begin{gathered} \left( {12\left( {R^{2} - \chi^{2} } \right)^{2} - 3\left( {R^{2} - \chi^{2} } \right)\left( { - 5r^{2} + 3R^{2} + 6\chi^{2} } \right)\ln \left( R \right) + 2\left( {r^{4} - 5r^{2} \chi^{2} + 4\chi^{4} } \right)^{2} \ln \left( R \right)^{2} } \right) \hfill \\ \left( {r^{2} - \chi^{2} } \right) - 6r^{2} \left( {R^{2} - \chi^{2} } \right)\ln \left( r \right)\left( {4\left( {R^{2} - \chi^{2} } \right) - \left( {2r^{2} - 3\left( {R^{2} + \chi^{2} } \right)} \right)\left( {\ln \left( {\frac{\chi }{R}} \right)} \right)} \right) - \hfill \\ \left( \begin{gathered} 3\left( {R^{2} - \chi^{2} } \right)\left( {5r^{4} + 3\chi^{2} \left( {R^{2} + 2\chi^{2} } \right) - r^{2} \left( {11R^{2} + 3\chi^{2} } \right)} \right) + 2 \hfill \\ \left( {2^{6} - 8\chi^{6} - 6r^{4} \left( {R^{2} + \chi^{2} } \right) + 9r^{2} \left( {R^{4} + \chi^{4} } \right)} \right)\ln \left( R \right) \hfill \\ \end{gathered} \right) \hfill \\ \ln \left( \chi \right) + 2\left( {\left( {r^{3} - 3rR^{2} } \right)^{2} - 4\chi^{6} } \right)\ln \left( \chi \right)^{2} \hfill \\ \end{gathered} \right) $$

$$ E(r) = \left( \begin{gathered} - \left( {r^{2} - \chi^{2} } \right)\left( {4\left( {R^{2} - \chi^{2} } \right) + \ln \left( R \right)\left( {5r^{2} - 7\chi^{2} + 4\left( {r^{2} - \chi^{2} } \right)\ln \left( R \right)} \right)} \right) \hfill \\ + 4r^{2} \ln \left( r \right)\left( {2\left( {R^{2} - \chi^{2} } \right) + \left( {r^{2} - 2\chi^{2} } \right)\left( {\ln \left( R \right) - \ln \left( \chi \right)} \right)} \right) + \hfill \\ \left( {5r^{4} + 7\chi^{4} - 4r^{2} \left( {2R^{2} + \chi^{2} } \right) + 4\left( {r^{4} - 2r^{2} \chi^{2} + 2\chi^{4} } \right)\ln \left( R \right)} \right)\ln \left( \chi \right) - 4\chi^{4} \ln \left( \chi \right)^{2} \hfill \\ \end{gathered} \right) $$

## Graphical outcomes and analysis

The temperature and hemodynamic velocity have been calculated using the hybrid nano-bloodstream model. The dynamical features of wall shear stress, resistance impedance, and pressure rise are characterized. Specifically, the effects of fractional parameter $\alpha$, relaxation time parameter $\lambda_{1}$, Grashof number $G_{r}$, heat source parameter $Q$, nanoparticles volume frictions $\phi_{1}$ and $\phi_{2}$, nanoparticles shape factor $n$, catheter’s maximum height parameter $\sigma$, and catheter’s radius parameter $e$ are examined. A graphical examination is performed to analyze the stenosis section (*a* > 0) and the aneurysm section (*a* < 0). To calculate the resistance impedance and pressure rise, numerical integration has been performed.

### Temperature

**Figure 2:**
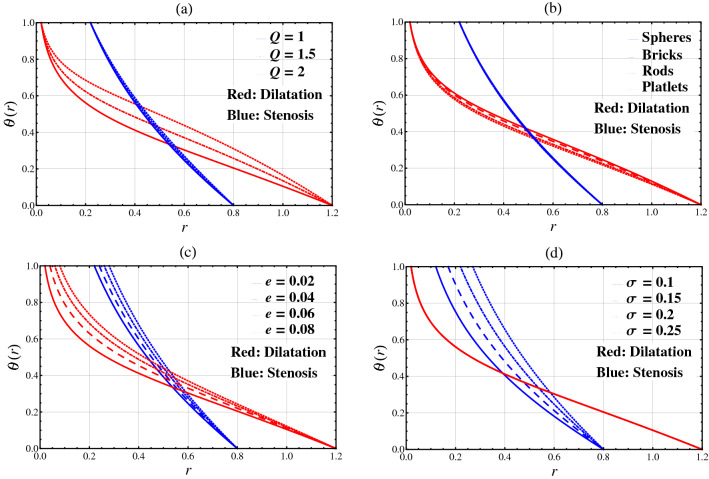
Temperature $\theta (r)$ against the radial coordinate $r$.

The temperature against the radial coordinate $r$ has been plotted for different values of heat source parameter $Q$, nanoparticle shape factor parameter *n*, catheter’s radius parameter $e$, and catheter’s maximum height parameter $\sigma$ in Fig. 2. Comparison is performed between stenotic and aneurysmal segments. It is seen from Fig. 2a,c that heat flow rises in both segments as the heat source parameter $Q$ and the catheter’s radius parameter $e$ increase. Figure 2b shows that the temperature rises for spherical-shaped nanoparticles in both regions. It is observed from Fig. 2d that the temperature drops by increasing the balloon catheter’s maximum height parameter $\sigma$ in stenosis segment while remains unchanged in dilatation segment.

Physically, the changing in heat source parameter and nanoparticle shape factor parameter bring temperature variations. Thus, we observe the higher temperature variation in the central region of the diseased artery. While, in the vicinities of catheter and arterial walls, the temperature is autonomous around. Also, by changing the value of the catheter’s radius parameter, the temperature pattern changes near the catheter wall. In the vicinity of the stenosis/aneurysmal wall, however, the temperature is autonomous around**.** Moreover, by altering the balloon catheter’s maximum height parameter, the temperature pattern near the catheter wall in stenosis segment changes. While, near the wall of stenosis, the temperature is autonomous around.

### Hemodynamic velocity

**Figure 3:**
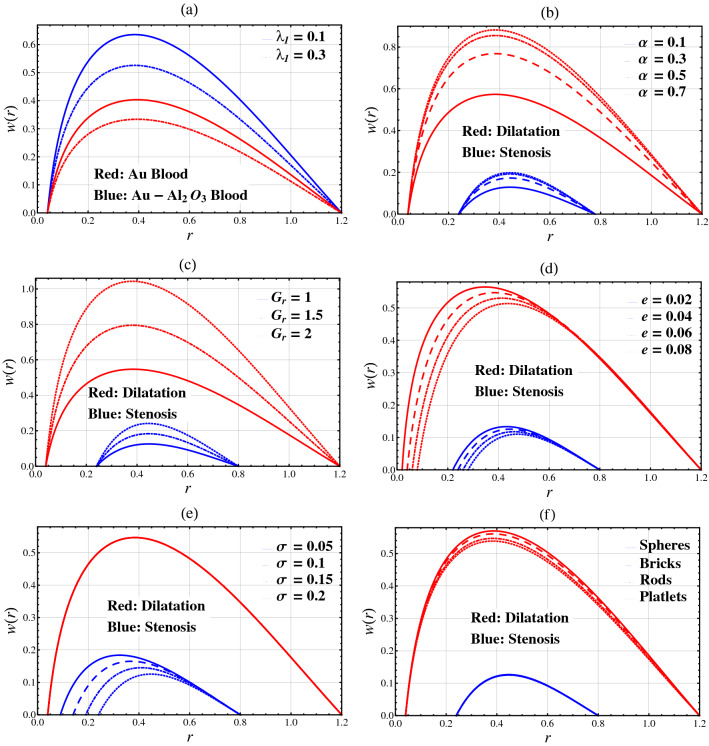
Hemodynamic velocity $w(r)$ against the radial coordinate $r$.

Figure 3 plots hemodynamic velocity against $r$ for different values of relaxation time parameter $\lambda_{1}$, fractional parameter $\alpha$, Grashof number parameter $G_{r}$, catheter’s radius parameter $e$, catheter’s maximum height parameter $\sigma$, and nanoparticle shape factor parameter *n*. In Fig. 3, comparisons are performed between hybrid nanofluid and nanofluid, and between stenosis and aneurysm. In Fig. 3a, it is discovered that the blood velocity for nanofluid is much lower than that of hybrid nanofluid. Also, by increasing the relaxation time parameter $\lambda_{1}$, the blood velocity drops. It is observed from Fig. 3b,c that the blood gains momentum upon increasing the fractional parameter $\alpha$ and the Grashof number parameter $G_{r}$. Figure 3d,f show that the blood velocity drops with respect to the catheter’s radius parameter $e$, while blood velocity is enhanced for spherical-shaped nanoparticles as compared to the other shapes of nanoparticle. Figure 3e shows that the blood velocity decreases upon increasing the catheter’s maximum height parameter $\sigma$ in the stenosis segment while remains unchanged in the dilatation segment. Moreover, it is observed that blood velocity in the stenosis segment is much lower than that of the dilatation segment.

Physically, by changing the relaxation time parameter, the fractional parameter, Grashof number and nanoparticle shape factor, we can see that the hemodynamic velocity in changing behavior between the catheter wall and arterial wall but near the wall of catheter and stenosis/aneurysm, the hemodynamic velocity is autonomous around. Also, by changing the value of the catheter’s radius parameter, the hemodynamic velocity is changing behavior near the catheter wall but near the arterial wall, the hemodynamic velocity is autonomous around. Moreover, by changing the values of balloon catheter’s maximum height parameter, the hemodynamic velocity is changing behavior near the catheter wall in the stenosis segment. However, near the wall of stenosis, the hemodynamic velocity is autonomous around.

### Wall Shear Stress (WSS)

**Figure 4:**
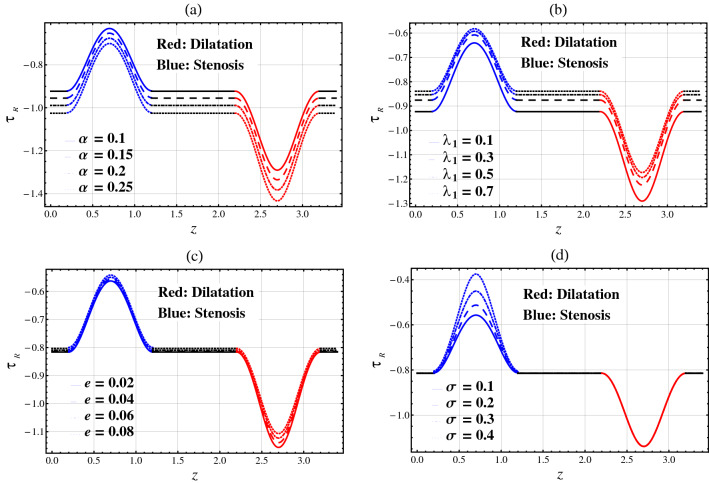
Wall shear stress $\tau_{R}$ against axial distance $z$.

**Figure 5:**
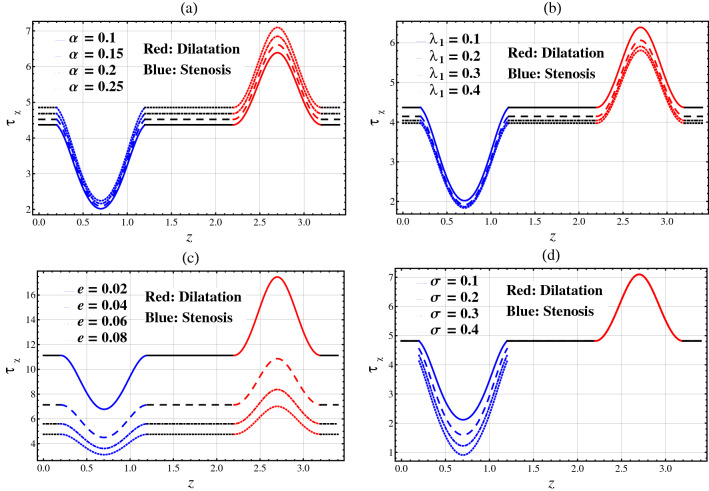
Wall shear stress $\tau_{\chi }$ against axial distance $z$.

The WSS is important to comprehend the movement of infection in the artery because of the connection between the restriction of arteriosclerosis (dilatation/stenosis) and catheter/arterial wall. Figure 4a–d are plotted to show the changes in WSS on arterial wall segment ($\tau_{R}$) for different parameters. Figure 5a–d show the changes in WSS on catheter wall segment ($\tau_{\chi }$) for different parameters. The shear stress at the catheter wall is higher than the arterial wall.

#### Arterial Wall Shear Stress ($\tau_{R}$)

In Fig. 4, for the stenotic segment, it is noted that $\tau_{R}$ ($\beta_{1}$ to $\beta_{1} + L_{0}$) increases towards the upstream of the stenotic section (i.e. $z$ = $\beta_{1}$) and peaks at $z = \beta_{1} + \frac{{L_{0} }}{2}$ before it drops and attains its minimal value at $z = \beta_{1} + L_{0}$. For the aneurysmal segment, $\tau_{R}$ ($\beta_{2}$ to $\beta_{2} + L_{0}$) decreases along the downstream direction of the aneurysmal section (i.e. $z$ = $\beta_{2}$) to reach its minimum at $z = \beta_{2} + \frac{{L_{0} }}{2}$, before it rises and peaks at $z = \beta_{2} + L_{0}$.

Figure 4 plots the arterial wall shear stress $\tau_{R}$ against $z$ for different values of fractional parameter $\alpha$, relaxation time parameter $\lambda_{1}$, catheter’s radius parameter $e$, and catheter’s maximum height parameter $\sigma$. Figure 4a shows that $\tau_{R}$ decreases with respect to the fractional parameter $\alpha$ in the whole arterial segment. Figure 4b,c show that $\tau_{R}$ increases with respect to the relaxation time parameter $\lambda_{1}$ and the catheter’s radius parameter $e$ in the whole artery. It is shown from Fig. 4d that $\tau_{R}$ increases with respect to the catheter’s maximum height parameter $\sigma$ in the stenosis segment. $\tau_{R}$ remains unchanged with respect to $\sigma$ in the whole artery except for the stenosis segment.

#### Catheter Wall Shear Stress ($\tau_{\chi }$)

In Fig. 5, for the stenotic segment, it is noted that $\tau_{\chi }$ ($\beta_{1}$ to $\beta_{1} + L_{0}$) experiences a sharp drop along the downstream direction of the stenotic section (i.e. $z$ = $\beta_{1}$) and bottoms at $z = \beta_{1} + \frac{{L_{0} }}{2}$ before it increases and peaks at the end of stenotic segment (i.e. $z = \beta_{1} + L_{0}$). For the aneurysmal segment, $\tau_{\chi }$ ($\beta_{2}$ to $\beta_{2} + L_{0}$) increases and peaks at $z = \beta_{2} + \frac{{L_{0} }}{2}$, before it drops and bottoms at $z = \beta_{2} + L_{0}$.

Figure 5 plots the catheter wall shear stress $\tau_{\chi }$ against $z$ for different values of fractional parameter $\alpha$, relaxation time parameter $\lambda_{1}$, catheter’s radius parameter $e$, and catheter’s maximum height parameter $\sigma$. Figure 5a shows that $\tau_{\chi }$ increases with respect to the fractional parameter $\alpha$ in the whole arterial segment. Figure 5b,c show that $\tau_{\chi }$ decreases with respect to the relaxation time parameter $\lambda_{1}$ and the catheter’s radius parameter $e$ in the whole artery. It is seen from Fig. 5d that $\tau_{\chi }$ increases with respect to the catheter’s maximum height parameter $\sigma$ in the stenosis segment. However, $\tau_{\chi }$ is independent to $\sigma$ in the whole artery except for stenosis segment.

### Resistance impedance

**Figure 6:**
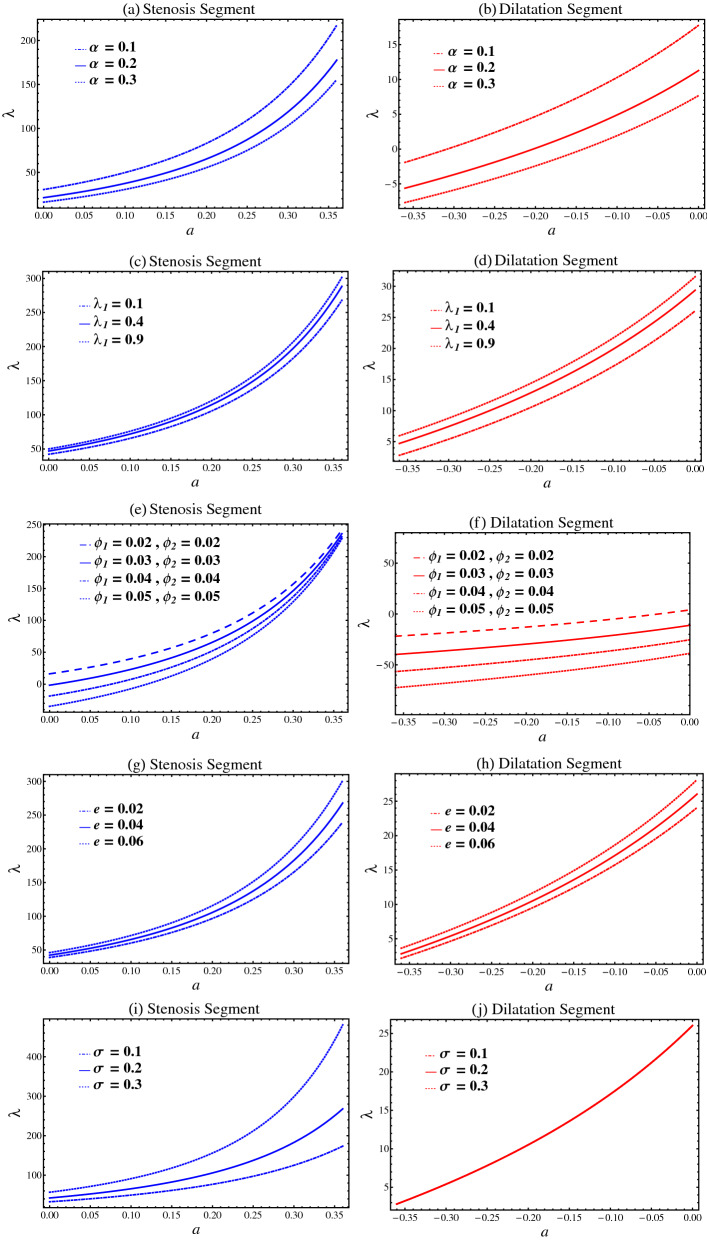
Resistance impedance $\lambda$ against $a$.

Figure 6 plots the resistance impedance $\lambda$ against the maximum height $a$ for stenosis and aneurysm in the presence of catheter. The resistance impedance $\lambda$ for different values of fractional parameter $\alpha$, relaxation time parameter $\lambda_{1}$, nanoparticles volume frictions $\phi_{1}$ and $\phi_{2}$, catheter’s radius parameter $e$, and catheter’s maximum height parameter $\sigma$ is examined. For both stenosis and aneurysmal segments, it is discovered that resistance impedance $\lambda$ is inversely proportional to $a$. It is also worth noting that for maximal $a$, the resistance impedance $\lambda$ for stenosis is higher than that for aneurysm. From Fig. 6a,b,e,f, it is observed that by increasing the fractional parameter $\alpha$ and nanoparticles volume frictions $\phi_{1}$ and $\phi_{2}$, the resistance impedance $\lambda$ decreases. Physically, the resistance impedance would drop upon increasing the fractional parameter and nanoparticles volume frictions. Figure 6c,d,g,h show that the resistance impedance $\lambda$ increases by increasing the relaxation time parameter $\lambda_{1}$ and the catheter’s radius parameter $e$. Physically, the increases in relaxation time parameter and catheter’s radius parameter can enhance the resistance impedance. It is seen from Fig. 6i,j that by increasing the catheter’s maximum height parameter $\sigma$, the resistance impedance $\lambda$ increases in stenosis segment and it remains unchanged in the dilatation segment. It is also observed that the resistance impedance for the stenosis segment is higher as compared to the dilatation segment.

### Trapping

**Figure 7:**
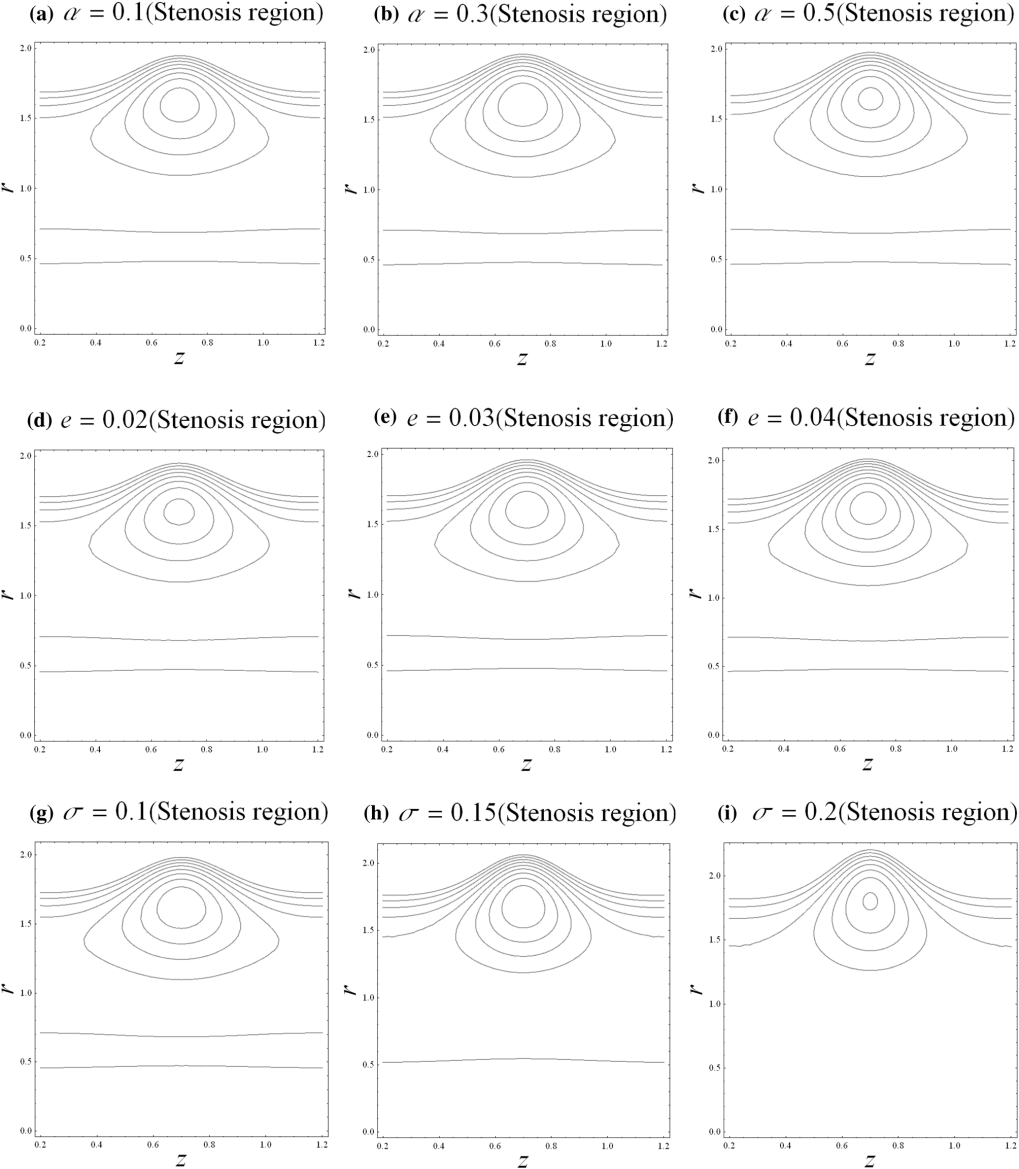
Streamlines with $z$ in stenosis segment.

**Figure 8:**
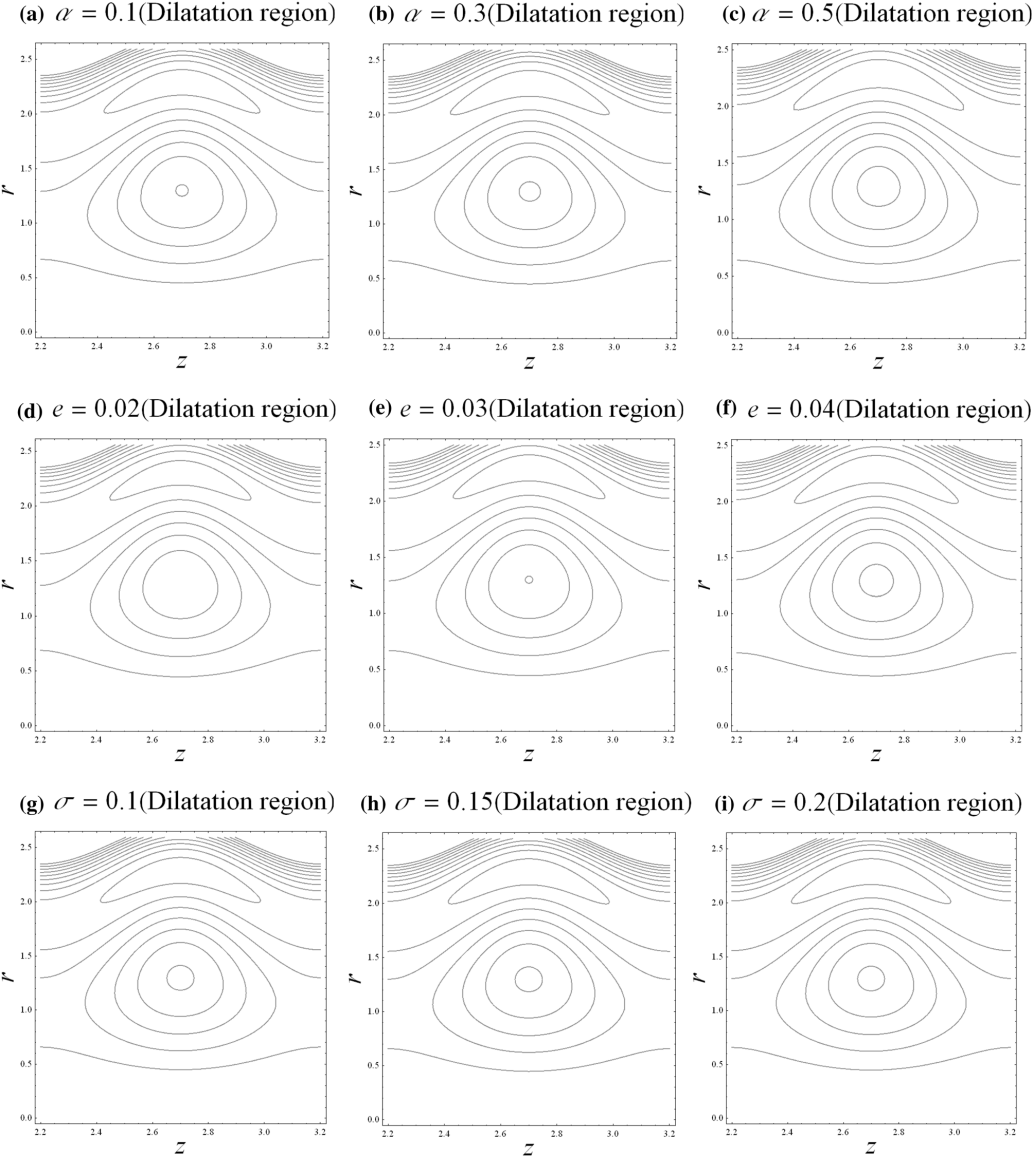
Streamlines with *z* in dilatation segment.

Trapping is significant as it is related to the hydrodynamic characteristics of aneurysm and stenosis segments. Contour plot is the best way to visualize the trapping process. The trapping mechanism is visualized through the revolving bolus internally. The streamlines are enclosed by the scale of the flowing bolus in the fluid. The trapping mechanism for the related parameters such as fractional parameter $\alpha$, catheter’s radius parameter $e$, and catheter’s maximum height parameter $\sigma$ is discussed for both stenosis and aneurysm segments. It is observed from Fig. 7 that the circulation of rotated bolus becomes larger in size upon increasing the fractional parameter $\alpha$ and catheter’s radius parameter $e$ while the circulation of rotated bolus becomes smaller in size upon increasing the catheter’s maximum height parameter $\sigma$ in the stenosis segment. It is found from Fig. 8 that the circulating bolus size increases with respect to the fractional parameter $\alpha$ and the catheter’s radius parameter $e$ in the dilatation segment. It is also observed that the size of the circulation bolus is independent to the catheter’s maximum height parameter $\sigma$ in the dilatation segment. Generally, the increases in fractional parameter and catheter’s radius parameter would enhance the circulating bolus size.

## Conclusion

In this paper, the fractional second-grade, non-isothermal, hybrid nanofluid model is used to study the blood flow characteristics in a concentrically catheterized, stenosed and aneurysmal arterial segments. The exact solutions are obtained and the effects of the relevant parameters are examined. The key discoveries are:

- The temperature is enhanced for the case of spherical-shaped nanoparticles as compared to other shapes of nanoparticle.
- Temperature is directly proportional to the heat source.
- As compared to nanofluid, the hemodynamic velocity of hybrid nanofluid is higher.
- Blood velocity rises for the case of spherical-shaped nanoparticles as compared to the other nanoparticle shapes.
- Blood velocity for the dilatation segment is higher than that of the stenosis segment.
- The arterial wall shear stress in the stenosis segment increases to its maximum before it drops significantly and bottoms at the end of the stenosis segment, whereas the opposite trend of arterial wall shear stress is detected in the aneurysmal segment as compared to the stenosis segment.
- The catheter wall shear stress in the aneurysmal segment climbs to its highest value and then begins to decrease steeply towards the end of the aneurysmal segment, whereas the opposite trend of catheter wall shear stress is detected in the stenotic segment as compared to the aneurysmal segment.
- Opposite behavior of wall shear stress is observed on arterial and catheter wall segments.
- Wall shear stress on the catheter wall segment is more than that of the arterial wall segment.
- For stenosis, the resistance impedance is higher in the aneurysm segment.
- The trapping mechanism depicts the formation of a rotating bolus in the stenotic and aneurysmal segments with varying parameters.
